# Urinary Calprotectin loses specificity as tumour marker due to sterile leukocyturia associated with bladder cancer

**DOI:** 10.1371/journal.pone.0213549

**Published:** 2019-03-14

**Authors:** Kathrin Bausch, Elisa Roth, Stefan Heinz, David Horst, Susanne Mathia, Tatjana Vlajnic, Lukas Bubendorf, Timm Westhoff, Christian Wetterauer, Hans Helge Seifert, Jan Ebbing

**Affiliations:** 1 Urological University-Clinic Basel-Liestal, University Hospital Basel, Basel, Switzerland; 2 University of Basel, Basel, Switzerland; 3 Department of Pathology, University Hospital Charité Berlin, Berlin, Germany; 4 Department of Nephrology, University Hospital Charité Berlin, Berlin, Germany; 5 Institute of Pathology, University Hospital Basel, Basel, Switzerland; 6 Department of Nephrology, Marien Hospital Herne, University Clinic of the Ruhr-University Bochum, Herne, Germany; University of Washington, UNITED STATES

## Abstract

**Background:**

Urinary Calprotectin, a mediator of the innate immune system, has been identified as a biomarker in bladder cancer. Our aim was to investigate the association between sterile leukocyturia and urinary Calprotectin in low-grade and high-grade bladder cancer.

**Materials and methods:**

We performed a prospective cross-sectional study including 52 patients with bladder cancer and 40 healthy controls. Definition of sterile leukocyturia was > 5.0 leukocytes per visual field in absence of bacteriuria.

**Results:**

The rate of sterile leukocyturia in low-grade (60.0%) and high-grade (62.0%) bladder cancer was comparable (p = 0.87). However, the median absolute urinary leukocyte count in patients with sterile leukocyturia was significantly higher in high-grade than in low-grade bladder cancer (p < 0.01). Spearman correlation revealed a significant correlation between urinary Calprotectin and leucocyte concentration (R = 0.4, p < 0.001). Median urinary Calprotectin concentration was 4.5 times higher in bladder cancer patients with than in patients without sterile leukocyturia (p = 0.03). Subgroup analysis revealed a significant difference in urinary Calprotectin regarding the presence of sterile leukocyturia in high-grade patients (596.8 [91.8–1655.5] vs. 90.4 [28.0–202.3] ng ml-1, p = 0.02).

Multivariate analysis identified the leukocyte concentration to be the only significant impact factor for urinary Calprotectin (OR 3.2, 95% CI 2.5–3.8, p = 0.001). Immunohistochemistry showed Calprotectin positive neutrophils and tumour cells in high-grade bladder cancer with sterile leukocyturia.

**Conclusions:**

Urinary Calprotectin cannot be regarded as a specific tumour marker for bladder cancer, but rather as a surrogate parameter for tumour inflammation.

## Introduction

Bladder cancer (BC) is the most common malignancy of the male and female urinary system, with urothelial carcinoma being the predominant histologic type in developed countries [1]. For primary diagnostics and during surveillance, international guidelines recommend invasive methods like cystoscopy and transurethral resection of suspicious lesions to obtain histology for a final exclusion of malignancy [2]. Voided urine cytology is recommended as an adjunct to cystoscopy to detect high-grade tumours. Over the past years, research efforts were directed towards finding less invasive alternatives, such as biomarkers, that reflect the presence of urothelial carcinoma, which is considered an indicator for a high risk of both low-grade and high-grade tumours.

Proteomic studies in blood serum of patients with BC revealed Calprotectin–the heterodimer of the proteins S100A8 and S100A9 –as a tumour-associated protein that is linked to bladder wall muscle invasion of the tumour as well as cancer-specific survival [3,4]. On the other hand, S100A8 and S100A9 are predominantly released by neutrophils and monocytes [5]; therefore, blood serum levels are affected by a number of conditions, including any kind of inflammation, which limits the implementation of these approaches in serum in daily clinical practice [6]. In a retrospective study, Ebbing et al. [7] were able to show that BC was associated with increased levels of urinary Calprotectin (uC). A cut-off value of 140 ng ml-1 resulted in a sensitivity and specificity of 80.4% and 92.5%, respectively. uC concentrations even differentiated between low-grade and high-grade BC [7]. Since neutrophils associated to urinary tract infections may release Calprotectin, Ebbing et al. [7] identified uC as a potential biomarker for BC under the exclusion of urinary tract infections. However, immunohistochemical findings indicate that Calprotectin is produced by both the tumour cells per se and myeloid cells that infiltrate the tumour, such as neutrophils [7]. Therefore, the influence of a sterile leukocyturia (SL) on uC concentrations remains unclear.

Our study aimed to investigate both the association between SL and uC levels and the immunohistochemical expression of Calprotectin in patients with low-grade or high-grade BC regarding the presence of SL, as compared to a healthy control group.

## Materials and methods

### Protocol

We performed a cross-sectional study with prospectively collected data at the Urological University Clinic Basel-Liestal of the University Hospital Basel, Switzerland. We consecutively enrolled 52 patients with histologic evidence of urothelial cancer of the bladder treated with a transurethral resection of a bladder tumour (TURBT) as well as additional 40 patients that served as healthy controls (no bladder tumour and no leukocyturia). The population of healthy controls has already been involved in studies that were published previously [7,8].

Exclusion criteria were acute deterioration of the renal function (acute kidney injury (AKI)), any bacteriuria in urine culture, prior renal transplantation, previous BCG (Bacillus Calmette-Guerin)-treatment, and secondary TURBT [9].

In order to measure the urinary biomarker Calprotectin, to obtain a urine culture and urinalysis to exclude a bacteriuria, and to measure the leukocyte counts per visual field (further referred to as leukocyte concentration), all participants provided urine samples (10 ml) directly after admission–prior to TURBT, either from a mid-stream specimen of urine or from a one-time bladder catheterisation. Definition of leukocyturia was > 5.0 leukocytes per visual field [10]. Serum creatinine was measured and estimated glomerular filtration rate (eGFR) was calculated according to the MDRD formula [11]. In the BC group, classification and grading of the resected bladder tumour was performed according to the TNM (UICC 2017) staging system and the 2004 WHO grading system.

The study was approved by the local Ethics Committee (Ethikkommission Nordwest- und Zentralschweiz) Basel, Switzerland (EKNZ). All study participants signed written informed consent.

### Measurement of Calprotectin concentrations in the urine

The urine samples were stored frozen (-20°C) until the assessment of Calprotectin. The urine concentrations of Calprotectin were quantified using an enzyme-linked immunosorbent assay (ELISA) kit (PhiCal Calprotectin, catalogue number K6927+urine buffer, Immundiagnostik AG, Bensheim, Germany), as previously described [7,8]. We refrained from adjusting Calprotectin concentrations to urinary creatinine, as we have previously shown that this adjustment does not lead to a further increase of accuracy [9].

### Immunohistochemistry

For immunohistochemistry, 5 μm tissue sections were deparaffinised and stained on a Leica Bond III autostainer, or manually by retrieving antigens in TRS6 (Dako Cytomation) for 20 minutes in a microwave oven [12]. Primary antibodies used for incubation were rabbit polyclonal anti-MPO (myeloperoxidase) (Thermo Scientific, 1:150) and biotinylated chicken polyclonal antibody against the S100A8/A9 heterocomplex (antibody chicken anti-MRP8/14 (mouse, rat), catalogue number A 1098.2, Immundiagnostik AG, Bensheim, Germany). Next, we employed a signal amplification technique based on streptavidin, biotin, and peroxidase [7]. Mouse spleen served as positive control, whereas omission of the primary antibody in serial sections served as negative control. Staining was visualized by Bond Polymer Refine Detection (Leica) or Vectastain ABC kits (Vector labs). Slides were counterstained with hematoxylin (Vector labs).

### Statistical analysis

Comparisons of uC concentrations and of leukocyturia (SL and leukocyte concentration) between the groups (BC group vs. healthy control group; low-grade BC vs. high-grade BC, groups with SL vs. groups without SL) were performed by means of a Mann-Whitney U test. Correlations were calculated using a non-normality Spearman’s rank correlation coefficient to determine the association between uC and leukocyte concentration. A linear regression test and a multivariant analysis were applied to define independent prognostic factors for the increase of uC. All tests were performed at a significance level of α = 0.05. Continuous data are shown as median with interquartile range. All analyses were performed with SPSS Statistics 19 (SPSS Inc., Chicago, Illinois, USA) and Graph Pad Prism Version 6.0 (GraphPad Software, La Jolla, California, USA).

## Results

We enrolled 92 subjects, including 52 BC patients and 40 healthy controls. The epidemiological data of the BC patients, concomitant disease, renal parameters, and data on the malignancy are depicted in Table 1. uC assessment was successful in every enrolled subject.

**Table 1:** Tumour patients' characteristics.

	Tumour patients
	(*n* = 52)
*Epidemiology*	
Female	12 (23.1%)
Male	40 (76.9%)
Age (years)	74 (39–92)
Body mass index (kg/m^2^)	26.1 (17.1–45.3)
*Concomitant diseases*	
Diabetes mellitus	11 (21.2%)
Hypertension	30 (57.7%)
Preexisting CKD	15 (28.8%)
Coronary heart disease	13 (25.0%)
*Medication on admission*	
ACE-I./ARB	17 (32.7%)
Diuretics	12 (23.1%)
*Urothelial carcinoma*	
Low grade	15 (28.8%)
High grade	33 (63.5%)
G1	7 (13.5%)
G2	16 (30.8%)
G3	25 (48.1%)
pTa	30 (57.7%)
pT1	7 (13.5%)
pT2	6 (11.5%)
pT3a	1 (1.9%)
pT3b	1 (1.9%)
pT4	0 (0%)
concomitant pCIS	19 (36.5%)
pure pCIS	2 (3.8%)
*Renal data*	
eGFR (ml min per 1.73m2-1)	71.0 (42.5–89.75)
creatinine on admission (mg dl-1)	0.85 (0.72–1.21)

CKD, chronic kidney disease; ACE-I./ARB, Angiotensin converting enzyme inhibitor/angiotensin receptor blocker; eGRF, estimated glomerular filtration rate.

**Fig 1:**
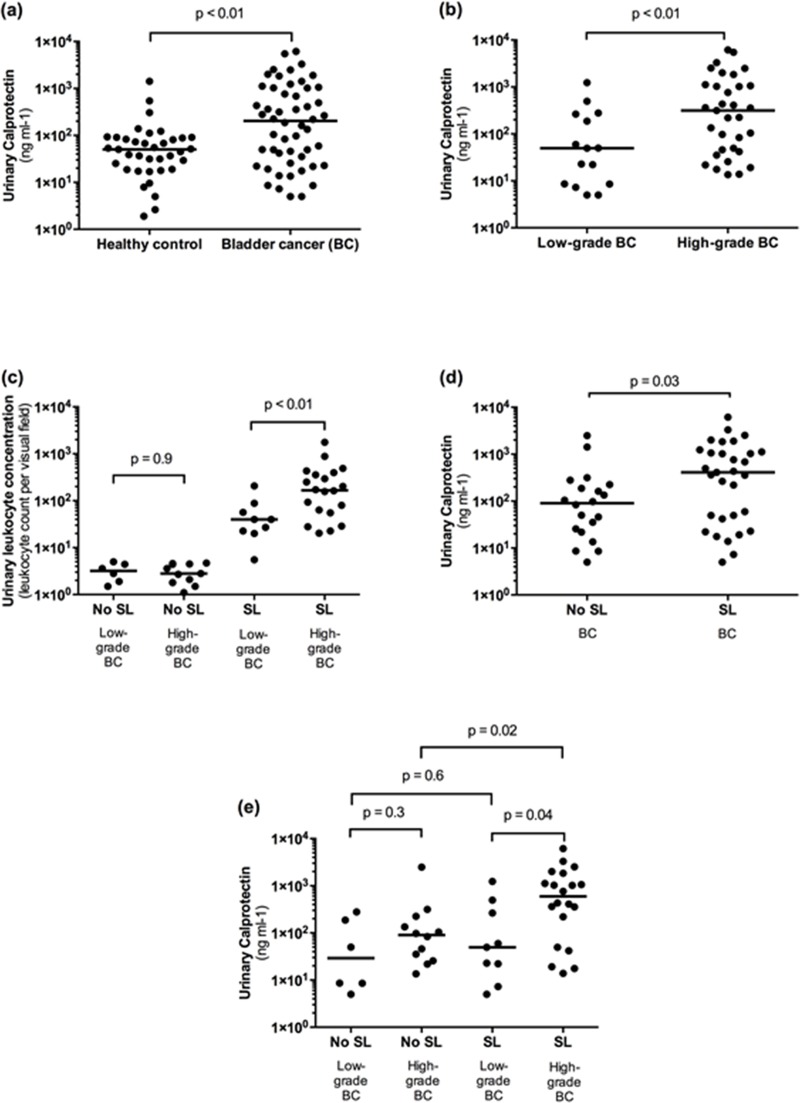
Urinary Calprotectin (uC) in bladder cancer (BC) and its association with sterile leukocyturia (SL) **(a)** Median uC levels in BC were significantly higher than in healthy controls (203.0 [28.0–1022.6] ng/ml vs. 51.0 [20.5–86.6] ng/ml). **(b)** Patients with high-grade BC had a significant higher median uC concentration than patients with low-grade BC (315.0 [43.9–1087.6] ng/ml vs. 49.4 [8.6–264.9] ng/ml. **(c)** The median absolute urinary leukocyte concentration was significantly higher in high-grade BC than in low-grade BC (167.3 [57.1–386.1] vs. 39.9 [21.4–72.2]). **(d)** Median uC concentration was 4.5 times higher in BC patients with SL (408.8 [42.0–1117.0] ng/ml) than in patients without SL (90.4 [22.7–215.4] ng/ml). **(e)** Subgroup analysis revealed a significant difference in uC concentration regarding the presence of SL in patients with high-grade BC (596.8 [91.8–1655.5] ng/ml vs. 90.4 [28.0–202.3] ng/ml), but not with low-grade BC (49.4 [14.7–379.7] ng/ml vs. 29.2 [7.7–210.0] ng/ml).

Median (IQR) uC concentration in patients with BC was four times higher than in healthy controls (203.0 [28.0–1022.6] ng ml-1 vs. 51.0 [20.5–86.6] ng ml-1, p < 0.01; Fig 1(A)). Median (IQR) uC concentration in patients with high-grade BC was significantly higher compared to patients with low-grade BC (315.0 [43.9–1087.6] ng ml-1 vs. 49.4 [8.6–264.9] ng ml-1, p = 0.01; Fig 1(B)). The overall rate of SL in patients with BC was 60.8%, with equal rates in patients with low-grade (60%) and high-grade (62%) BC (p = 0.87). However, the median (IQR) absolute leukocyte concentration was significantly higher in high-grade BC than in low-grade BC (167.3 [57.1–386.1] ng ml-1 vs. 39.9 [21.4–72.2] ng ml-1; p < 0.01; Fig 1(C)).

**Fig 2:**
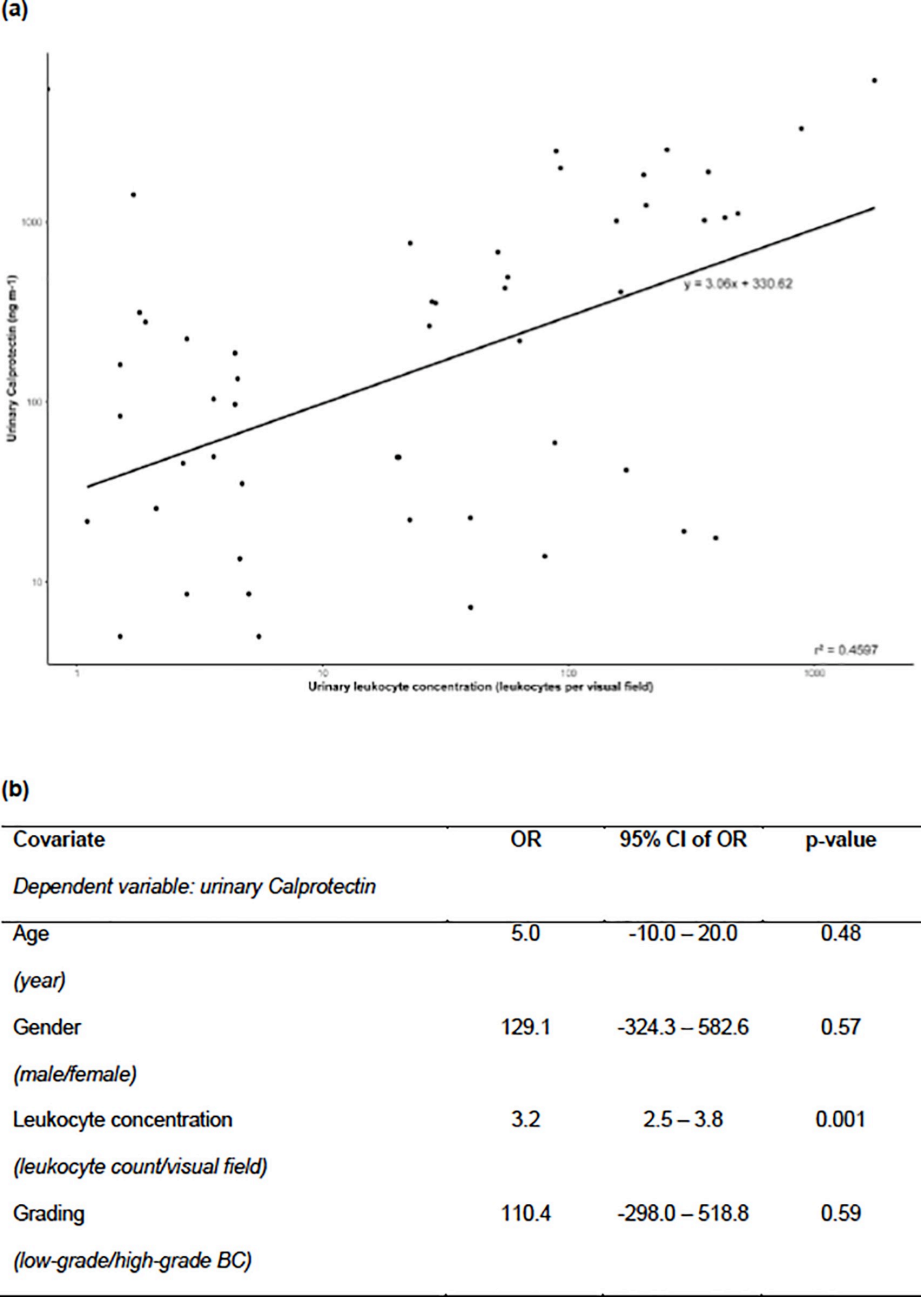
Correlation of urinary Calprotectin (uC) and sterile leukocyturia (SL). **(a)** Regression line is plotted. Linear regression model: R = 0.68, B = 3.06, (95% CI 2.12–4.00), p<0.001. Spearman`s correlation: R = 0.4 (95% CI 0.14–0.62), p = 0.001. **(b)** Multivariant regression analysis identified leukocyte concentration to be the only significant impact factor for uC concentration. B, regression co-efficient, slope of regression line; BC, bladder cancer, CI, confidence interval; OR, odds ratio; R, correlation co-efficient; r^2^, goodness of fit.

We found a significant correlation between uC concentration and urinary leukocyte concentration in patients with BC (R = 0.4, 95% CI 0.014–0.62, p = 0.001). This association was confirmed by a linear regression model (R = 0.68, B = 3.06, 95% CI 2.12–4.00, p < 0.001; Fig 2(A)). Median (IQR) uC concentration was 4.5 times higher in BC patients with SL (408.8 [42.0–1117.0] ng ml-1) than in patients without SL (90.4 [22.7–215.4] ng ml-1, p = 0.03; Fig 1(D)).

Subgroup analysis of BC patients with SL revealed a significant difference in median (IQR) uC level between low-grade and high-grade BC (49.4 [14.7–379.7] ng ml-1vs. 596.8 [91.8–1655.5] ng ml-1, p = 0.04; Fig 1(E)). Furthermore, subgroup analysis showed a statistically significant difference in median (IQR) uC concentration regarding the presence of SL in patients with high-grade BC (596.8 [91.8–1655.5] ng ml-1 vs. 90.4 [28.0–202.3] ng ml-1, p = 0.02), but not with low-grade BC (49.4 [14.7–379.7] ng ml-1 vs. 29.2 [7.7–210.0] ng ml-1, p = 0.6; Fig 1(E)). Subgroup analysis of BC patients without SL revealed no significant difference in median (IQR) uC level between low-grade and high-grade BC (29.2 [7.7–210.0] ng ml-1 vs. 90.4 [28.0–202.3] ng ml-1, p = 0.3; Fig 1(E)).

A multivariate analysis identified leukocyte concentration to be the only significant impact factor for uC concentration (OR 3.2, 95% CI 2.5–3.8, p = 0.001); age (years) (OR 5.0, 95% CI -10.0–20.0, p = 0.48), gender (male/female) (OR 129.1, 95% CI -324.3–582.6, p = 0.57), and grading (low-grade/high-grade) (OR 110.4, 95% CI -298.0–518.8, p = 0.59) did not reach statistical significance (Fig 2(B)).

**Fig 3:**
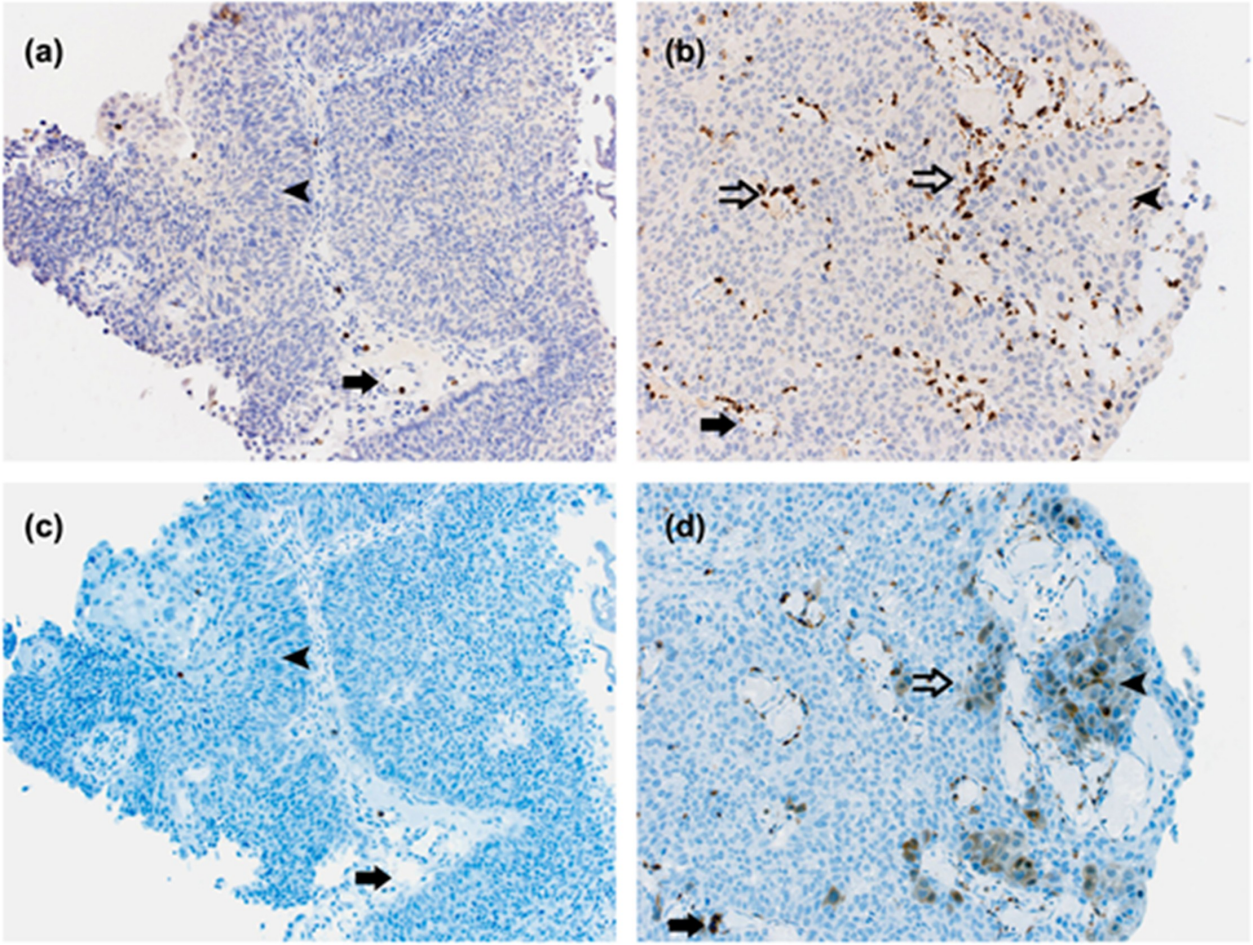
Immunostaining for MPO **(a,b)** and Calprotectin (**c**, **d**) in urothelial carcinoma (TNM [UICC 2017] pTaG3 high-grade without SL [**a**, **c**] and pTaG2 high-grade with SL [**b**, **d**]). The antibodies are specific for Calprotectin and MPO; solid arrow: intra-capillary neutrophil cell; open arrow: infiltrating neutrophil cell; arrowhead: tumour cell. In high-grade BC without SL less neutrophils are shown in the capillaries (**a**, solid arrow) which are negative for Calprotectin (**c**, solid arrow) whereas in high-grade BC with SL capillaries are densely filled (**b**, solid arrow) and are positive for Calprotectin. In BC without SL neutrophils do not invade the tumour (**a**, **c**). In BC with SL neutrophils invade the tumour (**b**, open arrow) and feature cytoplasmatic staining of Calprotectin (**d**, open arrow). Tumour cells in high-grade BC without SL are Calprotectin negative (**a**, **c**, arrow head), whereas tumour cells in high-grade BC with SL show Calprotectin staining (**b**, **d**, arrow head). Not all tumour cells in high-grade BC with SL are Calprotectin positive. MPO, myeloperoxidase, SL, sterile leukocyturia.

Next, we located Calprotectin protein with help of immunohistochemistry in archival paraffin-embedded tumours from 12 patients (3 patients: pTa low-grade BCs without SL; 3 patients: pTa low-grade BCs with SL; 3 patients: pTa high-grade BCs without SL; and 3 patients: pTa high-grade BCs with SL). Sensitivity and specificity of the antibody were tested in human paraffin-embedded spleen. In low-grade and high-grade BCs with and without SL, capillaries were filled with myeloid cells with fragmented nuclei and were hence neutrophils that partially showed cytoplasmic staining of Calprotectin. In high-grade BC tumour with SL, neutrophils invaded the tumour (Fig 3B and 3D), whereas high-grade BC without SL showed non-such infiltration (Fig 3A and 3C)). In high-grade BC with SL, neutrophils invading the tumour expressed Calprotectin, and BC tumour cells featured Calprotectin staining (Fig 3B and 3D), whereas in high-grade BC without SL, the tumour cells did not show cytoplasmic staining of Calprotectin (Fig 3A and 3C). In the demonstrated example (pTa G3 high-grade without SL and pTa G2 high-grade with SL), uC were 21.76 ng ml-1 and 3309.15 ng ml-1, and leukocyte concentrations were 1.1 and 883.5 per visual field, respectively. In the high-grade BC group with SL, parts of the tumour were negative for Calprotectin. In low-grade BCs with and without SL, neutrophils invading the tumour as well as Calprotectin positive neutrophils and tumour cells were very rare.

## Discussion

Our results question the potential of uC as a specific tumour marker in urothelial BC on one hand, and clarify the correlation between uC and SL concentrations in the urine as well as the association between tumour infiltration of neutrophils and tumour associated Calprotectin expression on the other. Median uC levels in BC were significantly higher than in the healthy controls, and a significant difference in uC concentrations between low-grade and high-grade tumours could be demonstrated. However, median uC levels in the presented overall BC group and in both low-grad and high-grade BC subgroups were lower compared to a different series that had been published previously (overall BC groups: 203.0 ng ml-1 vs. 522.3 ng ml-1, low-grade BC subgroups: 49.4 ng ml-1 vs. 351.9 ng ml-1, and high-grade BC subgroups 315.0 ng ml-1 vs. 1635.2 ng ml-1) [7].

Increasing experimental and clinical evidence suggests that changes in the expression and function of S100 proteins play a crucial role during cancer development: S100A8 and S100A9 up-regulation in serum was described in breast, lung, gastric, colorectal, pancreatic, and prostate cancer, as well as in BCs [6, 13]. In addition, solid cancers are not only autonomous masses of transformed tumour cells, but also consist of multiple cell types, including adjacent fibroblasts and epithelial cells, innate and adaptive immune cells, as well as cells from the blood and lymphatic vasculature, which create a tumour-specific microenvironment [13]. Elevated S100A8 and S100A9 protein levels are a hallmark of numerous pathological conditions associated with inflammation (e.g. rheumatoid arthritis, systemic lupus erythematosus, giant cell arteritis, cystic fibrosis, and inflammatory bowel diseases) [14, 15]. In summary, accumulating evidence suggests that high S100A8/S100A9 levels, in their function as a danger-associated molecular pattern, are characteristic for inflammatory conditions, and that they act as chemotactic molecules constitutively expressed by neutrophils, activated monocytes, and macrophages [15,16] that promote further leukocyte recruitment [17].

Assessment of Calprotectin–the heterodimer of S100A8/S100A9 d–in the urine offers the opportunity to investigate the urinary tract selectively. Thus, uC predominantly reflects alterations in the inflammatory urinary tract microenvironment. However, although a similar rate of SL in low-grade and high-grade BC (60% and 62%)—defined as > 5.0 leukocytes per visual filed—was found in our study, a significantly higher rate of urinary leukocyte concentration was demonstrated in high-grade compared to low-grade BC.

BC as well is a highly immunogenic malignancy [17], and an impact of host immune response has been described on tumour development and progression [18]. The microenvironment in BC tissue resembles a status of chronic inflammation and the immune response in cancer is ensured by macrophages, granulocytes, and lymphocytes [19]. In particular, granulocyte-type CD15(high) CD33(low) cells and monocyte-type CD15(low) CD33(high) cells were found in BC tissue and produced substantial amounts of proinflammatory chemokines, and interfere with cancer cells as well as with the T-cell system, thereby regulating the invasive potential of cancer cells and the functional efficiency of the local immune response [20]. Those cells are the key players in the production of Calprotectin [5].

Therefore, we performed both a Spearman correlation and a linear regression model, which revealed a significant correlation between uC concentration and leukocyte concentration in patients with BC. Consequently, uC concentration in BC patients with SL was significantly 4.5-fold higher compared to uC concentration in patients without SL. Subgroup analysis revealed a significant difference in uC concentrations regarding the presence of SL in patients with high-grade BC, but not with low-grade BC, emphasizing the high influence of leukocyte concentration on urinary calprotectin levels. When investigating the BC subgroup without SL, a significant differentiation in uC concentrations between high-grade and low-grade BC could subsequently no longer be observed. Therefore, we conducted a multiple linear regression analysis that confirmed the urinary leukocyte concentration to be the only significant impact factor for uC concentration, whereas age, gender, and tumour grading did not reach statistical significance.

A proinflammatory microenvironment in BC was recently also found by Ebbing et al., who demonstrated a dense staining with Calprotectin antibody in urothelial cytoplasm as well as in intravascular leukocytes infiltrating the tumour [7]. These findings were specified by our immunohistochemical investigations: low-grade BCs with and without SL show only little staining of Calprotectin in either neutrophils or tumour cells. Neutrophils in high-grade BC without SL show almost no invasion into the tumour, and tumour cells are predominantly negative for Calprotectin staining, whereas in high-grade BC with SL, tumour cells are frequently infiltrated by neutrophils, which are partly positive for Calprotectin representing the proinflammatory microenvironment of the tumour. Furthermore, in high-grade BC with SL even tumour cells express Calprotectin. Therefore, higher uC in high-grade compared to low-grade BC might be the result of multiplication effects of tumour infiltration Calprotectin positive neutrophils and tumour cells also expressing Calprotectin.

Consequently, a multitude of further conditions affect uC; recent results show that acute kidney injury leads to increased concentrations of uC [9]. Furthermore, urinary tract infections come along with increased uC concentrations due to the elevated levels of neutrophils [9]. Finally, BCG- or intravesical chemotherapies induce local inflammation and therefore preclude the use of uC for surveillance in these patients.

Even though studies on the prognostic value of inflammatory biomarkers in BC have been published since the 1970s [21–23], a recent systematic meta-analysis (including 34 mostly retrospective publications) on the use of inflammatory biomarkers in BC revealed that none of the markers has proven to be sufficiently useful for clinical application due to a wide heterogeneity of impact factors on these biomarkers [24].

If uC loses specificity as a tumour marker for BC due to SL, the immune dependent expression of Calprotectin might be useful in a different way.

While surgery activates stress-sensing pathways, necrosis of cancer cells is promoted by chemotherapy, immunotherapy, and radiation therapy. In BCG therapy–as an example–neutrophil leukocytes play a primary immune role and account for 75% of the cells recruited to the bladder following instillation [25]. This highly inflammatory process results in the release of several mediators of the immune system, including danger associated molecular patterns such as Calprotectin. Besides the regulation of inflammatory processes, apoptosis induction in malignant cells is a primary function of Calprotectin [26]. It remains unclear, however, whether the inflammation induced by these therapies supports or inhibits future tumour development. One theory suggests that necrotic inflammation may stimulate the remaining malignant cells promoting cancer recurrence. Alternatively, this inflammation may deter cancer regrowth improving outcomes [27]. While this is an area where future research could advance and improve current therapies like immunotherapy, uC might serve as a predictor for treatment response and relapse in the context of the inflammatory microenvironment in BC: In a previous study in BC patients investigating serum levels of S100A8 and S100A9 –the monomers of Calprotectin—its concentrations have already been shown to decrease postoperatively after TURBT [4]. Furthermore, an abnormal expression of the monomers was associated with an increased risk of disease progression and cancer-specific death [4].

Moreover, S100A8-related genes may be used as a molecular signature for the prediction of disease progression in non-muscle-invasive BCA [6]. Finally, a differential expression of S100A8 has been postulated to identify patients at high risk of being metastasized, even at a clinically localised stage in BCA [28]. All of these approaches, however, bear limitations that will probably impede an implementation in daily clinical practice. First, serum S100A8 and S100A9 levels are affected by a multitude of conditions, including any kind of inflammation and other malignant entities. Thus, an increase or decrease in plasma concentrations is very unspecific. In contrast, the assessment of Calprotectin in the urine has the advantage that this medium contains information predominantly related to the urinary tract, and might therefore, be comparable to the predictive value of faecal Calprotectin in monitoring disease activity, response to therapy, and predicting relapse in inflammatory bowel disease [29]. However, our study should be interpreted with consideration of its limitations related to the small sample size. Further studies are needed to clarify the role of uC as a diagnostic and therapeutic target in bladder carcinoma.

## Conclusion

uC in BC is a robust result of tumour infiltration by leukocytes, with high-grade BC showing higher sterile urinary leukocyte concentrations correlating with higher uC concentrations. In contrast to the preliminary results, uC cannot be regarded as a specific tumour marker for BC, but rather as a surrogate parameter for tumour inflammation.
